# Supplementary material for: Stoichiometric balance of protein copy numbers is measurable and functionally significant in a protein-protein interaction network for yeast endocytosis
Source: PLoS Comput Biol. 2018 Mar 8;14(3):e1006022. doi: 10.1371/journal.pcbi.1006022 (PMC5860782; doi:10.1371/journal.pcbi.1006022)
Supplement: S1 Text — (PDF) [file pcbi.1006022.s001.pdf]

Supporting Information Text for

Stoichiometric balance of protein copy numbers is measurable and functionally significant in a protein-protein interaction network for yeast endocytosis

David O Holland[1] and Margaret E Johnson[2*]

Supporting Text 1: Model details for the nine-protein vesicle forming module

Model Overview: The purpose of simulating the vesicle forming module was to determine the relative effects of copy number imbalance on clathrin recruitment and vesicle formation at the cell membrane. Our model utilized nine base species: the clathrin heavy chain (CHC1) in trimer form, clathrin light chain (CLC1), the lipid PtdIns(4,3)P_2, and the adaptor proteins ENT1/2, SLA2, YAP1801, YAP1802, SYP1, and EDE1. ENT1 and ENT2 were combined into one specie with a concentration defined by the sum of their individual copy numbers because their partners and binding affinities were equivalent. Parameters with notes and references are shown in Table S1. Steps such as triggering by cargo or the role of actin in vesicle invagination and scission were not captured.

Spatial Compartments: Spatial resolution was not characterized. Instead, the cytosol and membrane were treated as two separate compartments. The solution volume and the membrane surface area were defined based on the known dimensions of the yeast cell. For species and reactions that occurred in the membrane compartment, the concentrations and binding affinities are all converted from units of um^{-2} to standard solution units L^{-1} based on a single length-scale conversion, σ. The 'volume' of the membrane compartment is then given by Surface Area (SA)* 2σ. This parameter σ is not an arbitrary definition of the membrane depth. Rather, it is most accurately represented as the conversion between the equilibrium constants in 3D versus those in 2D, such that $K_a^{2D}=K_a^{3D}/(2\sigma)$. As we detailed in a recent study[1], σ is thus a thermodynamic property of each binding pair, and will depend on the molecular properties of the proteins involved. Although rarely measured, both experiment and theory[2-4] quantify σ on the nanometer lengthscale. In the NFsim simulations, all protein pairs will bind based on the same value of σ, which we set to 1 nm. The reduced dimensionality of the membrane surface, which is here captured in the effective 'volume' of the membrane compartment, creates higher concentrations of proteins on the membrane that then promotes binding. Binding events where one protein is in the cytosol and one protein on the membrane requires a search within the solution compartment for the

membrane bound protein. Thus, these reactions are based on the true 3D cytosolic volume to define the K_a for the reaction.

Vesicle formation reaction: Vesicle formation was treated as instantaneous once an aggregate of 100 triskelia was formed. A triskelia in our model was required to contain a heavy chain trimer bound to 3 light chains, otherwise it was not counted toward the sum. The aggregate was deleted at a rapid rate of 1000 s^-1 (NFSim does not allow a true instantaneous rule, but treats every rule as a type of reaction). Proteins were then added back to the cytosol at a rate of 1000 s^-1, until the total number of proteins equaled the starting number. In reality, it takes time for both the vesicle to scission -- with the help of the cell cytoskeleton -- and further time for the clathrin cage to be broken up and the proteins returned to the cytosol. But because our focus was on clathrin recruitment as it controls vesicle formation, and because parameters for protein recycling time are unavailable in the literature, we choose to make these rates uniformly rapid.

Binding affinities: Binding rates for human homologs were used when the binding rates for yeast proteins were not available. In the case of SYP1, a binding rate between a generic F-BAR domain and a PtdIns(4,5)P_2 molecule was used, but this assumes that the domain only binds one PtdIns(4,5)P_2 molecule when it may bind two. SYP1 may also bind to other types of lipids or to transmembrane "cargo" receptors. Thus the true binding rate may be stronger. But we found that increasing binding rate by a factor of 100 did not increase vesicle formation, because the limiting step was EDE1 binding to SYP1.

Structural assumptions: Steric hindrance may weaken or even prevent binding depending on the composition of the aggregate. For example, even though one SLA2 protein can bind one clathrin light chain, the composition of a SLA2 - triskelia complex may be less than 3 to 1 due to steric inhibition. But without further structural information, this steric inhibition is impossible to characterize. Similarly, EDE1 has three EH domains, but it is unclear how many partners it may bind at once. We provided EDE1 with two EH binding sites in our model.

Supporting Text References

1. Yogurtcu ON, Johnson ME. Cytoplasmic proteins can exploit membrane localization to trigger functional assembly. PLoS Comp Biol. 2018;Accepted.
2. Wu Y, Vendome J, Shapiro L, Ben-Shaul A, Honig B. Transforming binding affinities from three dimensions to two with application to cadherin clustering. Nature. 2011;475(7357):510-3. doi: 10.1038/nature10183. PubMed PMID: 21796210; PubMed Central PMCID: PMCPMC3167384.
3. Hu J, Lipowsky R, Weikl TR. Binding constants of membrane-anchored receptors and ligands depend strongly on the nanoscale roughness of membranes. Proc Natl Acad Sci U S A. 2013;110(38):15283-8. doi: 10.1073/pnas.1305766110. PubMed PMID: 24006364; PubMed Central PMCID: PMCPMC3780905.

4. Weikl TR, Hu J, Xu GK, Lipowsky R. Binding equilibrium and kinetics of membrane-anchored receptors and ligands in cell adhesion: Insights from computational model systems and theory. Cell Adh Migr. 2016;10(5):576-89. doi: 10.1080/19336918.2016.1180487. PubMed PMID: 27294442; PubMed Central PMCID: PMCPMC5079412.
