# Supplementary material for: Stoichiometric balance of protein copy numbers is measurable and functionally significant in a protein-protein interaction network for yeast endocytosis
Source: PLoS Comput Biol. 2018 Mar 8;14(3):e1006022. doi: 10.1371/journal.pcbi.1006022 (PMC5860782; doi:10.1371/journal.pcbi.1006022)
Supplement: S1 Table — (PDF) [file pcbi.1006022.s002.pdf]

S1 Table. Vesicle forming module rate parameters

Parameter	Description	Value	Notes and References
Vol_CP	Cytoplasm reaction volume	37.2 μm^3	60% of cell volume[1], for average of haploid cell volume (42 fL) and diploid cell volume (82 fL)[2]
SA_PM	Plasma membrane surface area	75.7 μm^2	Assuming a spherical cell, calculated from average of median haploid volume (42 fL) and diploid cell volume (82 fL)[2]
σ	Lengthscale conversion between K_a^{2D} and K_a^{3D}	1 nm	[3]
Kd_CHC_CHC	Clathrin heavy chain polymerization	100 µM	Order of. [4]
Kd_CHC_ENT	Clathrin heavy chain binding to ENT1/2	22 µM	Binding of amphiphysin peptide to clathrin box[5]
Kd_CHC_YAP	Clathrin heavy chain binding to YAP1801/2	160 µM	Based on AP180 binding in humans[6]
Kd_EDE_ENT	EDE1 to ENT1/2 binding	12 µM	Binding of EH domain to NPF motif. [7]
Kd_EDE_YAP	EDE1 to YAP1802 binding	0.6 µM	Binding of Eps15 to sAP180 in humans. Kd of 0.5-0.7 µM [8]
Kd_EDE_EDE	EDE1 dimerization	0.127 µM	[9]
Kd_CHC_CLC	Clathrin heavy chain to light chain binding	0.1 nM	Upper limit of binding strength to CHC1 trimers. [10]
Kd_CLC_SLA	Clathrin light chain to SLA2 binding	22 µM	Engqvist-Goldstein says that HIP1R (human homolog of SLA2) binds to clathrin cages with Kd in the low nanomolar range[11], but experiment was with clathrin cages rather than isolated light chains. We choose to assign same affinity as CHC1 to ENT1/2.[5]
Kd_SLA_SLA	SLA2 dimerization	1 nM	Arbitrarily strong rate chosen. For HIP1R (human homolog) virtually no monomers in vitro.[12]

Kd_SYP_SYP	SYP1 dimerization	2.5 μM	Rate based on FCHo2 (human homolog) self-binding.[13]
Kd_SYP_EDE	SYP1 to EDE1 binding	0.227 μM	[9]
Kd_L_ENT	ENT1/2 binding to lipid	0.02 μM	[14]
Kd_L_YAP	YAP1801 binding to lipid	0.3 μM	[14]
Kd_L_SLA	SLA2 binding to lipid	0.2 μM	[14]
Kd_L_SYP	SYP1 binding to lipid	53 μM	F-BAR domain binding to a single PIP_2 molecule.[15] Other papers suggest binding additional lipids or binding cargo, so may be stronger.
L_0	Density of lipid PtdIns(4,5)P2 partners	25,292 particles/ μm^2	Estimated from experiments with 3T3/NIH fibroblasts[16]
CHC1_0	Total clathrin heavy chain trimers	6426	19278 heavy chains[17]. Divide by 3.
CLC1_0	Total clathrin light chains	14538	[17]
EDE1_0	EDE1 total proteins	5964	[17]
ENT_0	ENT1/2 total proteins	3075	Sum of ENT1 and ENT2 proteins[17]
YAP1801_0	YAP1801 total proteins	357	[17]
YAP1802_0	YAP1802 total proteins	264	[17]
SLA2_0	SLA2 total proteins	3904	[17]
SYP1_0	SYP1 total proteins	2467	[17]
k_dump	Rate of deletion for a complex of >=100 triskelia	1000 s^{-1}	Arbitrarily high rate chosen
k_recyc	Rate of protein recycling to the cytoplasm	1000 s^{-1}	Arbitrarily high rate chosen

References:

1. Alberts B. Molecular biology of the cell. Sixth edition. ed. New York, NY: Garland Science, Taylor and Francis Group; 2015. 1 volume (various pagings) p.
2. Jorgensen P, Nishikawa JL, Breitkreutz BJ, Tyers M. Systematic identification of pathways that couple cell growth and division in yeast. Science. 2002;297(5580):395-400. doi: 10.1126/science.1070850. PubMed PMID: 12089449.
3. Yogurtcu ON, Johnson ME. Cytoplasmic proteins can exploit membrane localization to trigger functional assembly. PLoS Comp Biol. 2018;Accepted.

4. Wakeham DE, Chen CY, Greene B, Hwang PK, Brodsky FM. Clathrin self-assembly involves coordinated weak interactions favorable for cellular regulation. The EMBO journal. 2003;22(19):4980-90. doi: 10.1093/emboj/cdg511. PubMed PMID: 14517237; PubMed Central PMCID: PMCPMC204494.
5. Miele AE, Watson PJ, Evans PR, Traub LM, Owen DJ. Two distinct interaction motifs in amphiphysin bind two independent sites on the clathrin terminal domain beta-propeller. Nat Struct Mol Biol. 2004;11(3):242-8. doi: 10.1038/nsmb736. PubMed PMID: 14981508.
6. Zhuo Y, Ilangovan U, Schirf V, Demeler B, Sousa R, Hinck AP, et al. Dynamic interactions between clathrin and locally structured elements in a disordered protein mediate clathrin lattice assembly. J Mol Biol. 2010;404(2):274-90. doi: 10.1016/j.jmb.2010.09.044. PubMed PMID: 20875424; PubMed Central PMCID: PMCPMC2981644.
7. de Beer T, Hoofnagle AN, Enmon JL, Bowers RC, Yamabhai M, Kay BK, et al. Molecular mechanism of NPF recognition by EH domains. Nat Struct Biol. 2000;7(11):1018-22. doi: 10.1038/80924. PubMed PMID: 11062555.
8. Morgan JR, Prasad K, Jin S, Augustine GJ, Lafer EM. Eps15 homology domain-NPF motif interactions regulate clathrin coat assembly during synaptic vesicle recycling. J Biol Chem. 2003;278(35):33583-92. doi: 10.1074/jbc.M304346200. PubMed PMID: 12807910.
9. Boeke D, Trautmann S, Meurer M, Wachsmuth M, Godlee C, Knop M, et al. Quantification of cytosolic interactions identifies Ede1 oligomers as key organizers of endocytosis. Mol Syst Biol. 2014;10:756. doi: 10.15252/msb.20145422. PubMed PMID: 25366307; PubMed Central PMCID: PMCPMC4299599.
10. Winkler FK, Stanley KK. Clathrin heavy chain, light chain interactions. EMBO J. 1983;2(8):1393-400. PubMed PMID: 10872336; PubMed Central PMCID: PMCPMC555288.
11. Engqvist-Goldstein AE, Warren RA, Kessels MM, Keen JH, Heuser J, Drubin DG. The actin-binding protein Hip1R associates with clathrin during early stages of endocytosis and promotes clathrin assembly in vitro. J Cell Biol. 2001;154(6):1209-23. doi: 10.1083/jcb.200106089. PubMed PMID: 11564758; PubMed Central PMCID: PMCPMC2150824.
12. Wilbur JD, Chen CY, Manalo V, Hwang PK, Fletterick RJ, Brodsky FM. Actin binding by Hip1 (huntingtin-interacting protein 1) and Hip1R (Hip1-related protein) is regulated by clathrin light chain. J Biol Chem. 2008;283(47):32870-9. doi: 10.1074/jbc.M802863200. PubMed PMID: 18790740; PubMed Central PMCID: PMCPMC2583295.
13. Henne WM, Kent HM, Ford MG, Hegde BG, Daumke O, Butler PJ, et al. Structure and analysis of FCHo2 F-BAR domain: a dimerizing and membrane recruitment module that effects membrane curvature. Structure. 2007;15(7):839-52. doi: 10.1016/j.str.2007.05.002. PubMed PMID: 17540576.
14. Stahelin RV, Long F, Peter BJ, Murray D, De Camilli P, McMahon HT, et al. Contrasting membrane interaction mechanisms of AP180 N-terminal homology (ANTH) and epsin N-terminal homology (ENTH) domains. The Journal of biological chemistry. 2003;278(31):28993-9. doi: 10.1074/jbc.M302865200. PubMed PMID: 12740367.
15. Moravcevic K, Alvarado D, Schmitz KR, Kenniston JA, Mendrola JM, Ferguson KM, et al. Comparison of Saccharomyces cerevisiae F-BAR domain structures reveals a conserved inositol phosphate binding site. Structure. 2015;23(2):352-63. doi: 10.1016/j.str.2014.12.009. PubMed PMID: 25620000; PubMed Central PMCID: PMCPMC4319572.

16. Yoon Y, Lee PJ, Kurilova S, Cho W. In situ quantitative imaging of cellular lipids using molecular sensors. Nat Chem. 2011;3(11):868-74. doi: 10.1038/nchem.1163. PubMed PMID: 22024883; PubMed Central PMCID: PMCPMC3205457.
17. Kulak NA, Pichler G, Paron I, Nagaraj N, Mann M. Minimal, encapsulated proteomic-sample processing applied to copy-number estimation in eukaryotic cells. Nat Methods. 2014;11(3):319-24. doi: 10.1038/nmeth.2834. PubMed PMID: 24487582.
