# Supplementary figures and images for: Stoichiometric balance of protein copy numbers is measurable and functionally significant in a protein-protein interaction network for yeast endocytosis
Source: PLoS Comput Biol. 2018 Mar 8;14(3):e1006022. doi: 10.1371/journal.pcbi.1006022 (PMC5860782; doi:10.1371/journal.pcbi.1006022)

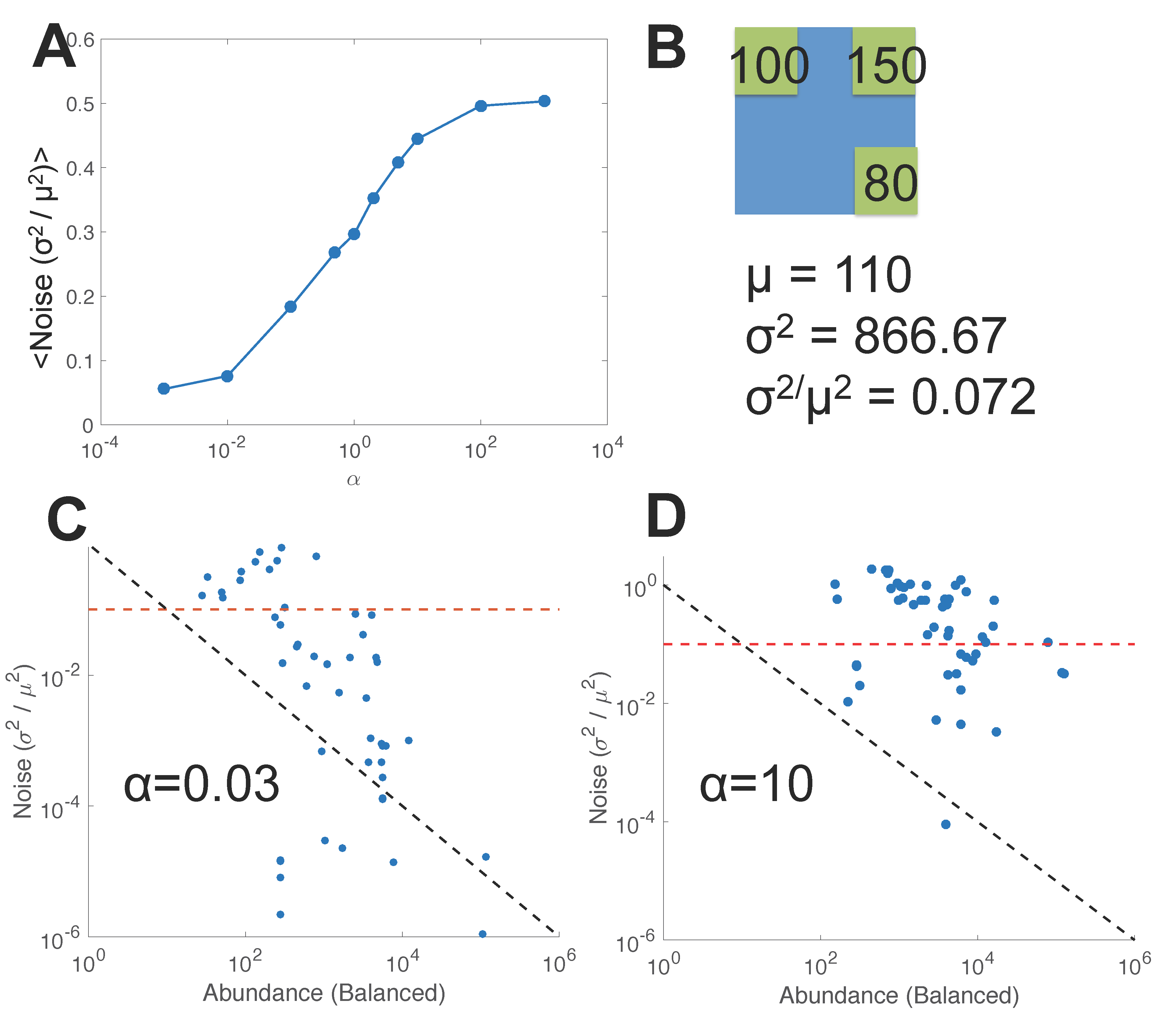

Supplement: S1 Fig — (A) Noise is calculated as the variance of the copy numbers assigned to interfaces on the same protein divided by the square of their average copy number. It does not refer to expression level noise. A high “α” parameter allowed greater variance, but even a low α could not remove noise entirely because there are no balanced solutions where all proteins can have interfaces of equal copy number. Noise had a sigmoidal relationship with log(α). (B) Example of interface noise on a protein. (C,D) Scatter plot of protein interface copy number noise vs a protein’s balanced “abundance”, the average of their interface copy numbers. The black line is where noise is inverse of abundance. The red line is noise = 0.1, which is expected to be the upper limit of noise when abundance exceeds ~1000 copy numbers [12]. For a low α, proteins varied widely in the amount of noise they have, though high-abundance proteins tended to have less noise, and were below the 0.1 threshold. As α was raised, proteins approached the same level of noise. (TIFF) [file pcbi.1006022.s004.tiff]

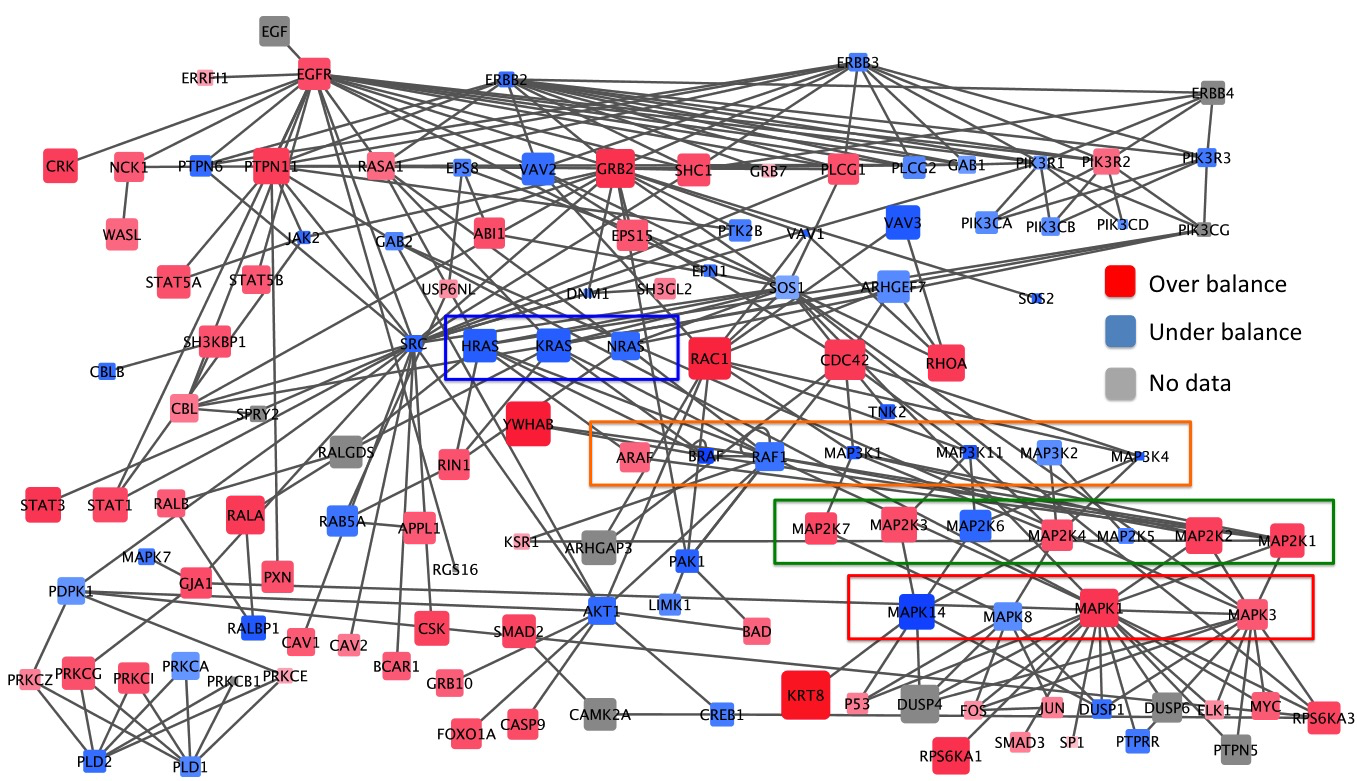

Supplement: S2 Fig — The ErbB network, which consists mainly of phosphorylation interactions, was not found to be statistically balanced based on the Jensen-Shannon divergence. However, certain proteins of note were found to be underexpressed, such as the three Ras proteins (HRAS, KRAS, and NRAS), and the MAP3K layer (RAF1, BRAF, ARAF, MAP3K1, MAP3K2, MAP3K4, and MAP3K11). Also underexpressed were the ErbB receptors and the hub SRC. These suggest a strategic imbalance of upstream proteins (in the case of MAPK cascades) or network bottlenecks (Ras proteins or SRC). Highlighted are the Ras proteins (blue), MAP3Ks (orange), MAP2Ks (green), and MAPKs (red). (TIFF) [file pcbi.1006022.s005.tiff]

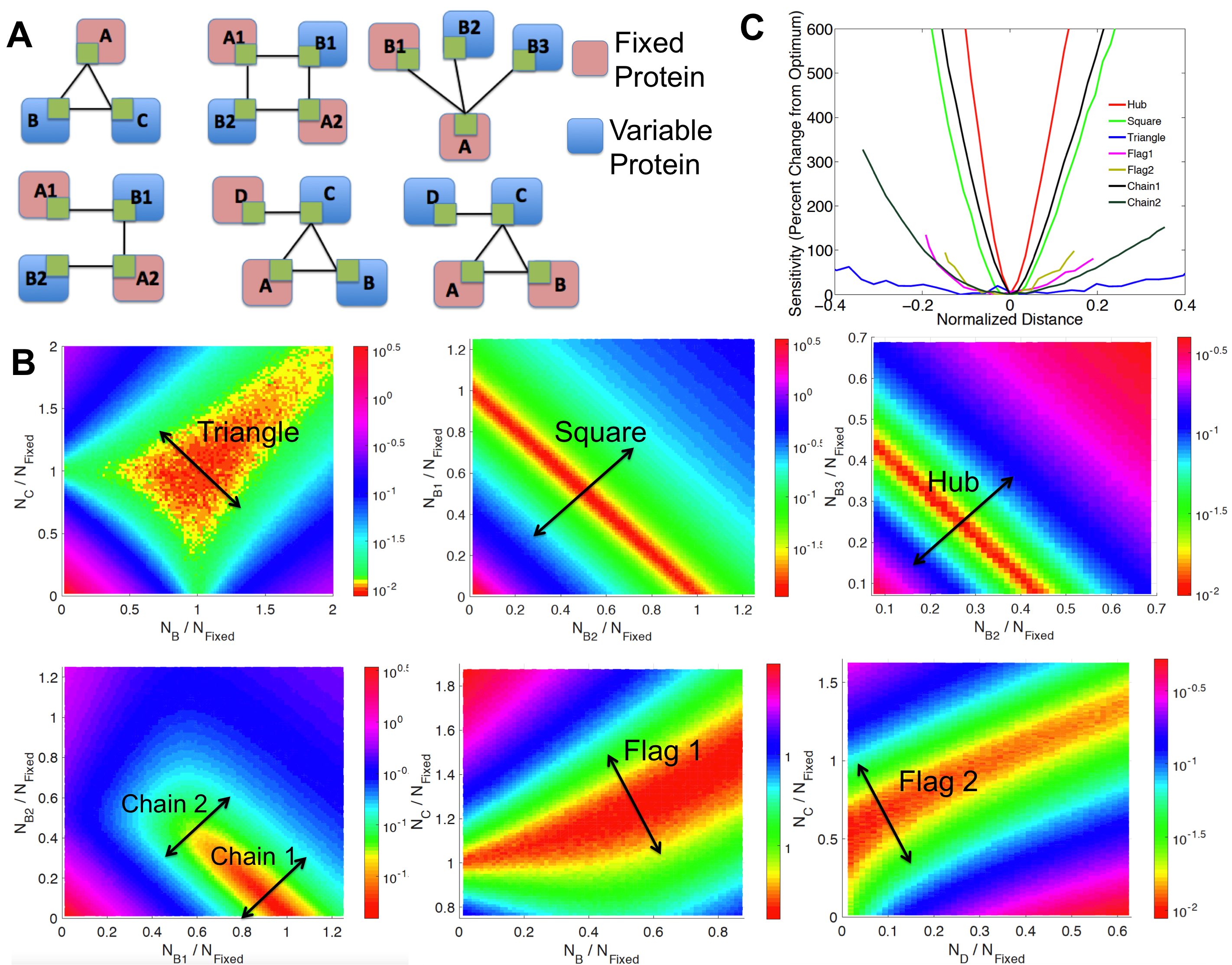

Supplement: S3 Fig — (A) Small networks used to construct the surface plots. For all simulations, two proteins had variable concentrations (blue) while the others had fixed concentrations (pink). (B) Surface plots of misinteraction frequency (color bar-Eq 1 main text). Misinteraction frequency is measured as Nnonspecific / (Nspecific + Nfree); that is, number of nonspecific complexes divided by all other species; at steady-state as described in the main text. Each plot corresponds to each respective network in A. The X and Y-axes are the concentrations of the variable proteins divided by the total concentrations of the fixed proteins. The black line is the principal component, which was used as an axis to measure the sensitivity of misinteractions as one moved away from a local minimum. For the chain we used two arbitrary local minima because the absolute minimum was when B2 = 0, a trivial solution. For the flag network we used two different sets of fixed and variable proteins because the surface plots were asymmetric. (C) The sensitivity of each network to misinteraction frequency as the protein concentrations moved away from an optimum (local minimum). Sensitivity is measured as percent change from the optimal (lowest) misinteraction frequency. (TIFF) [file pcbi.1006022.s006.tiff]

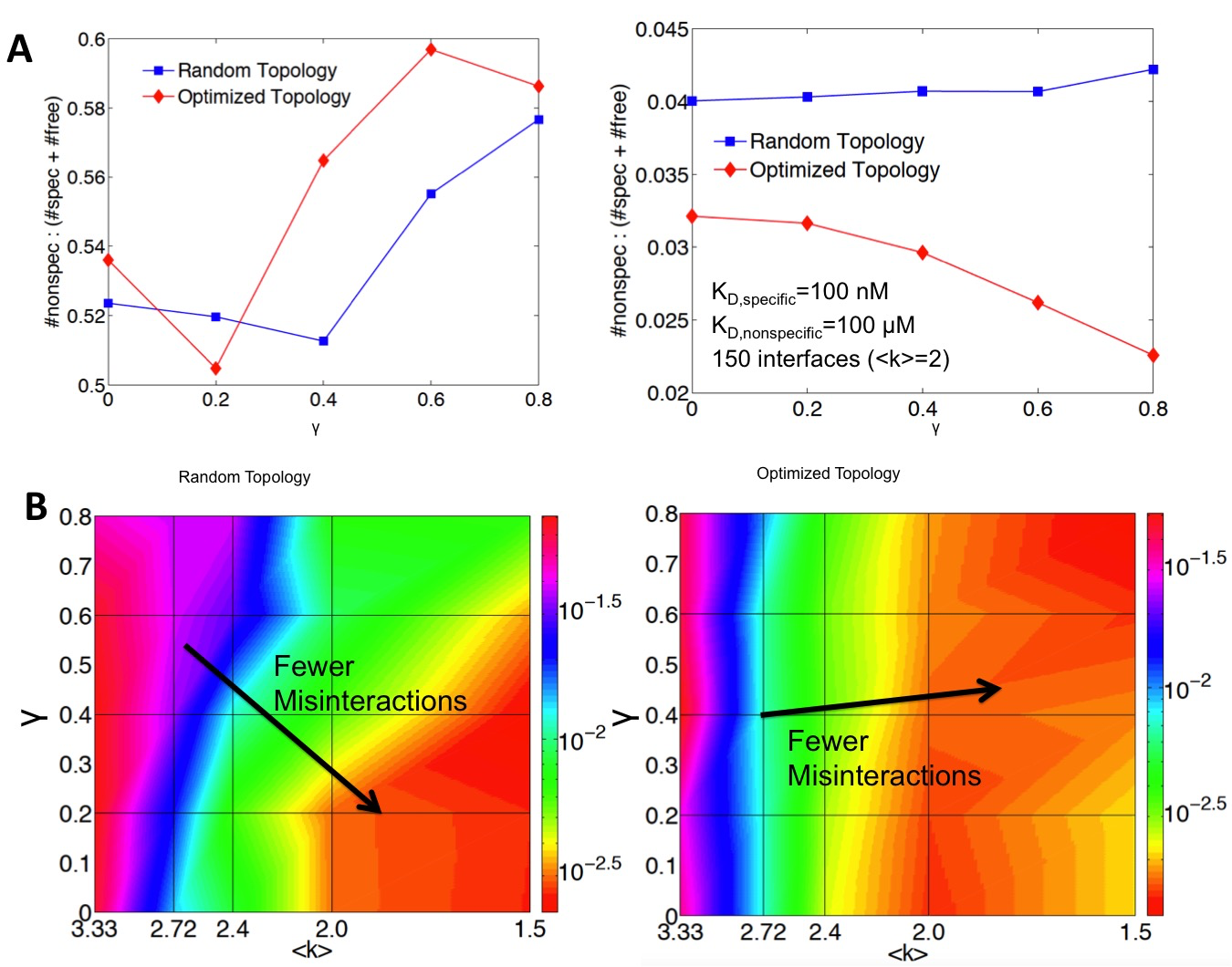

Supplement: S4 Fig — (A) Misinteraction frequency of networks under randomly sampled (left) and balanced copy numbers (right) when fixed energy gaps were used (KD, specific = 100nM, KD,nonspecific = 100μM). Networks with optimized topology and a power-law-like distribution (γ = 0.8) performed best under balanced copy numbers but worse under imbalance. (B) Heat map of misinteraction frequency under balanced copy numbers vs degree distribution and network density. Denser networks always had more misinteractions, but the effects of degree distribution depended on whether the local topology was optimized or not. (TIFF) [file pcbi.1006022.s007.tiff]

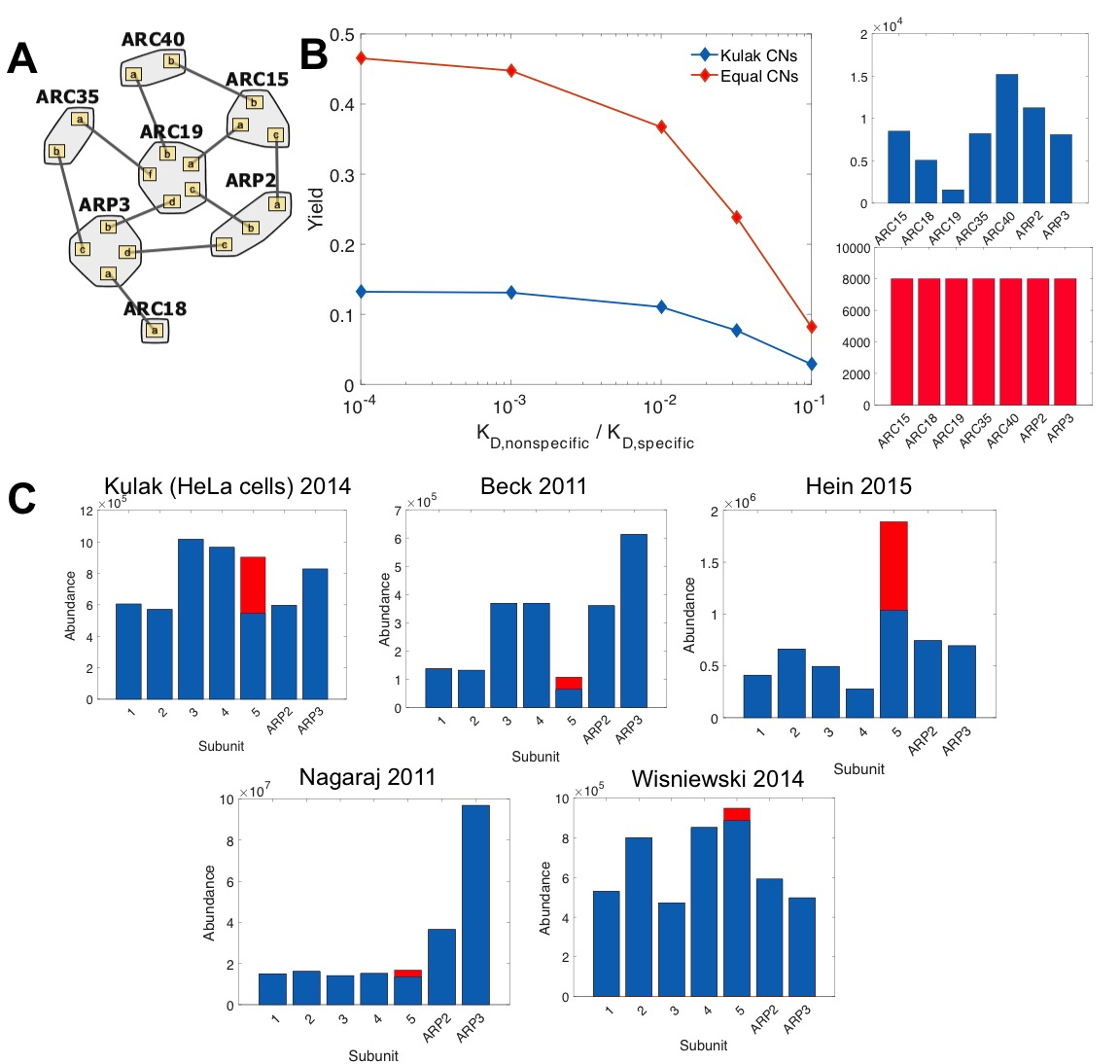

Supplement: S5 Fig — (A) Contact map of the seven subunits of the complex, generated with RuleBender{Smith, 2012 #488}(B) Under varying misinteraction strengths, the yield for the balanced copy numbers was always higher than for the observed copy numbers from Kulak et al.{Kulak, 2014 #276} Yield was measured as Ndesired / (Ndesired + Nundesired), which refer to the number of proteins in either desired (complete) complexes or undesired (incomplete or misassembled) complexes. (C) The observed copy number distribution was not found to be conserved between studies in either yeast or humans. Bar plots are from five studies of the ARP2/3 subunits in human cells. The red bar is for the addition of the “subunit 5-like” protein. Only one study (Hein et al.) found ARC19’s equivalent, subunit 4, to be underexpressed{Hein, 2015 #277}. (TIFF) [file pcbi.1006022.s008.tiff]
